# Modified Halloysite as an Adsorbent for the Removal of Cu(II) Ions and Reactive Red 120 Dye from Aqueous Solutions

**DOI:** 10.3390/molecules29133099

**Published:** 2024-06-28

**Authors:** Krzysztof Kuśmierek, Andrzej Świątkowski, Ewa Wierzbicka, Izabella Legocka

**Affiliations:** 1 Institute of Chemistry, Military University of Technology, 00-908 Warsaw, Poland; a.swiatkowski@wp.pl; 2 Department of Polymer Technology and Processing, Łukasiewicz-Industrial Chemistry Institute, 01-793 Warsaw, Poland; e.wierzbicka@outlook.com (E.W.); izabella.legocka@ichp.lukasiewicz.gov.pl (I.L.)

**Keywords:** adsorption, Reactive Red 120, copper ions, halloysite, chemical modification

## Abstract

The adsorption of copper ions and Reactive Red 120 azo dye (RR-120) as models of water pollutants on unmodified halloysite (H-NM), as well as halloysites modified with sulfuric acid (H-SA) and (3-aminopropyl)triethoxysilane (H-APTES), was investigated. The results showed that adsorption of both the adsorbates was pH-dependent and increased with the increase in halloysite dosage. The adsorption kinetics were evaluated and the results demonstrated that the adsorption followed the pseudo-second-order model. The adsorption isotherms of Cu(II) ions and RR-120 dye on the halloysites were described satisfactorily by the Langmuir model. The maximum adsorption capacities for the Cu(II) ions were 0.169, 0.236, and 0.507 mmol/g, respectively, for H-NM, H-SA, and H-APTES indicating that the NH_2_-functionalization rather than the surface area of the adsorbents was responsible for the enhanced adsorption. The adsorption capacities for RR-120 dye were found to be 9.64 μmol/g for H-NM, 75.76 μmol/g for H-SA, and 29.33 μmol/g for H-APTES. The results demonstrated that APTES-functionalization and sulfuric acid activation are promising modifications, and both modified halloysites have good application potential for heavy metals as well as for azo dye removal.

## 1. Introduction

Intense economic growth, accompanied by the development of urban areas, industrial zones, communication and transport systems, and increased production due to higher demand and consumption, contributes to increased environmental pressures. Economic development itself brings many opportunities and benefits. However, it is also a threat to the environment. Most of these pollutants (inorganic and organic) are harmful to both living organisms and humans. Thus, for example, most heavy metals are elements that are a burden to the organism, due to their high toxicity and their presence in the organism negatively affects its functioning [1,2]. Among the organic pollutants that are quite common in the environment, dyes, which are used on a large scale in the textile industry, are an important group. The first observable effect caused by dye contamination is the coloration of the water, which affects its aesthetic qualities as well as its transparency and sunlight permeability by interfering with photosynthesis processes. The presence of dyes in water impedes the flow of water through fish gills, impairing gas exchange, which contributes to reduced growth rates and even death. In addition, dyes cause a decrease in oxygen diffusion in water bodies, leading to animal extinctions. In addition, some classes of dye, particularly azo dyes, were shown to have carcinogenic and mutagenic properties [3,4,5].

Such negative effects of pollution on the environment and living organisms make it necessary to take appropriate measures to eliminate both the causes of pollution and its effects. The main technological processes for restoring water to its optimal parameters are classified into biological (aerobic and anaerobic treatment processes), chemical (ozonation, electrocoagulation, electrochemical, and oxidation processes), and physical (coagulation, flocculation, filtration, and adsorption) [2,5]. Each of these methods has its advantages and disadvantages. The various methods differ in their effectiveness in removing contaminants and in the cost of the process. Among all the methods, adsorption is undoubtedly one of the most popular techniques used in water treatment [2,5]. It is recommended because of its low cost, high efficiency, simplicity, and versatility, removing both organic and inorganic materials. Of course, the efficiency of adsorption is closely related to the physical and chemical properties of the adsorbent used. Hence, the selection of an appropriate adsorbent for the removal of specific contaminants is a priority challenge that will affect the overall efficiency of the process. The effectiveness of the water treatment process can be maximized by selecting an optimum adsorbent, i.e., one with the highest adsorption capacity at the lowest price. Finding new adsorbents is, therefore, very important. Many research groups are working on this.

Activated carbons are the most widely used adsorbents because their specific physicochemical properties (high porosity and specific surface area) result in their high adsorption capacity and high efficiency in removing aqueous pollutants. However, several processes and their complexity required to produce activated carbon, as well as the need for its periodic regeneration, affect its high cost, which significantly limits its use and application. This has stimulated many researchers to obtain and develop alternative low-cost adsorbents. In addition to low cost, such materials should of course have high availability and be either renewable or waste. Based on the classification proposed by Crini et al. [6], such non-conventional sorbents can be categorized into the following groups: (1) natural materials including inorganic and siliceous materials as well as clays; (2) agricultural wastes such as sawdust, bark, solid wastes (rice husk, nutshell, corn cob, wheat straw); (3) industrial by-products and wastes (fly ash, red mud, sludge); (4) biosorbents such as chitin/chitosan, peat, fungi or bacterial biomass; and (5) miscellaneous adsorbents, e.g., cotton waste, hydrogels.

A fairly common group of potential, low-cost sorbents are clays and other clay minerals [7,8,9,10], among which halloysite seems to be a very promising material. Halloysite belongs to the group of common and widely distributed minerals; its large deposits can be found in Poland, the USA, Brazil, China, Turkey, and elsewhere [11]. The morphology of the halloysite grain is related to its origin and especially to the crystallization conditions in the geological environment. Halloysite consists of a layer of silicon tetrahedra and aluminum octahedra that form a porous three-dimensional structure. Individual plates are separated from each other by a free space, called a gallery, in which there may be absorbed ions and molecules (including water) loosely bound to the surface of the plate using mainly hydrogen bonds. In the unit cell of the mineral, there are four hydroxyl groups associated with the octahedral layer, which have different orientations to the plane. Three of them are located on the outer surface of the bundle and one –OH group is located inside the bundle. Adsorption and grafting are two types of reactions that can occur on the surface of halloysite grains. Due to its physicochemical properties, halloysite can be used as a filler-modifier for a wide range of polymers [12], in the biological field [13], as an adsorbent to remove pollutants from water and wastewater [9,10]. In addition, it can be easily modified, e.g., by thermal treatment, acid activation, or functionalization, to tailor the adsorption properties of the mineral for a specific pollutant [9,10]. The effectiveness of both unmodified and modified halloysite as adsorbents for the removal of both heavy metals [10,14,15,16,17] and various dyes [10,18,19,20] from water was reported in numerous papers.

The purpose of this paper is to evaluate the suitability and effectiveness of unmodified halloysite (H-NM) as well as halloysites modified with sulfuric acid (H-SA) and (3-aminopropyl)triethoxysilane (H-APTES) as potential alternative adsorbents for the removal of water pollutants. The copper(II) ions and Reactive Red 120 azo dye (RR-120) were selected as models of water contaminants. The effect of the method used to modify the adsorbent on its ability to adsorb the metal as well as the dye was investigated. The adsorption kinetics and adsorption under equilibrium conditions, as well as the effects of adsorbent dosage and solution pH, were evaluated.

## 2. Results and Discussion

### 2.1. Characteristics of the Halloysite Samples

**Figure 1:**
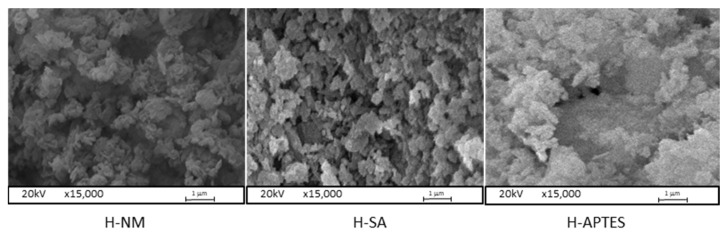
Scanning electron microscopy (SEM) images of the halloysite samples (magnification 15,000×).

SEM images of the raw and modified halloysite samples are shown in Figure 1 while the surface composition obtained from EDS analysis is summarized in Table 1.

The SEM images of the studied halloysite samples show its modification results. One can observe visible differences in the surface morphology between the original sample H-NM and modified samples H-SA and H-APTES. Treating H-NM with sulfuric acid increases its roughness. The deposition of APTES glued together halloysite particles.

The presence of carbon on the surface of the halloysite modified with APTES was observed. The carbon content in the H-APTES sample was 28.9 at.%, while no carbon was found in the H-NM and H-SA samples. This difference is due to the use of an organic compound in the modification process. The N observed in this sample is derived from APTES molecules. Modification of raw halloysite with sulfuric acid does not cause significant changes in the O, Al, Si, Fe, and Ti contents.

**Figure 2:**
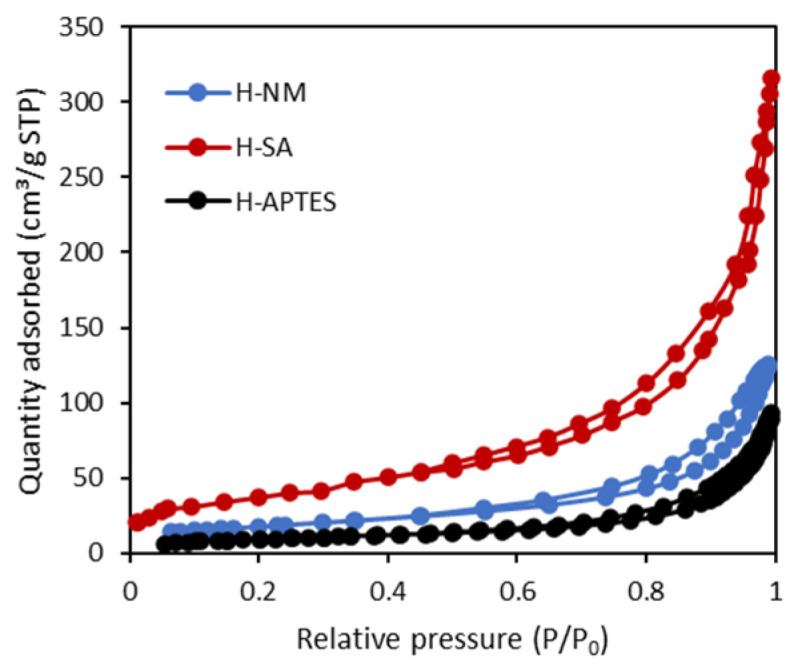
The nitrogen adsorption–desorption isotherms at 77 K for halloysite samples.

The porous structure of the halloysite samples was characterized using low-temperature nitrogen adsorption–desorption isotherms (Figure 2).

The specific surface areas (*S*_BET_) and pore volumes calculated from the N_2_ adsorption–desorption isotherms are listed in Table 2.

The porous structure of the halloysite samples was closely correlated with their modification methods. The use of sulfuric acid gave a significant increase in all porosity parameters. Their values were about 2 times greater for H-SA in comparison with the H-NM sample. For the halloysite modified with APTES observed effect was the opposite and values of porosity parameters were two times smaller for H-APTES in comparison with the H-NM sample.

The obtained from thermogravimetric analysis weight losses in characteristic temperatures (end of drying, maximum of DTG curves, end of weight losses) are listed in Table 3.

The first step of the mass loss associated with the increase in temperature concerns the removal of water (the dehydration of physisorbed water and interlayer water) in the case of H-NM and H-SA samples and in the case of H-APTES-ethanol. APTES is not removed due to its boiling point of 223 °C. Further steps of the TG curves include dehydroxylation (hydroxyl groups in the [AlO_6_] octahedral layer of halloysite are removed). All samples are at endothermic status during the whole heating process, attributed to continuous dehydration and dehydroxylation.

**Figure 3:**
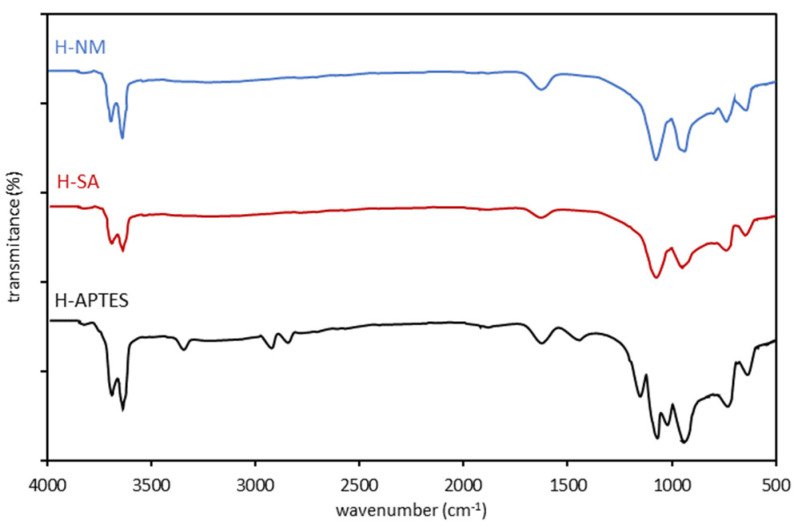
FTIR spectra of raw and modified halloysite samples.

The infrared spectra for H-NM, H-SA, and H-APTES halloysite samples are presented in Figure 3 and the specific absorption bands revealed in the FTIR vibration spectra in the range of 4000–400 cm^−1^ are listed for comparison in Table 4.

The infrared spectra of H-NM and H-SA halloysite samples show the following vibration bands: the bands at 3697 and 3622 cm^−1^, assigned to two Al_2_OH-stretching bands (vibrations of the inner OH and the OH located at the inner surface of halloysite tubes), the band at 1634 cm^−1^ which corresponds to strongly bending vibrations of the adsorbed water, the band at 1108 cm^−1^, assigned to apical Si–O, the band observed at 914 cm^−1^ caused by the O–H deformation of inner-surface hydroxyl groups, and O–H deformation of inner hydroxyl groups, the bands at 791 and 753 cm^−1^ assigned to O–H translation vibrations of halloysite O–H units. Compared to the spectrum of raw halloysite (H-NM) with acid-treated (H-SA), one can observe that the spectra of the modified sample show a significant decrease in the intensity of all bands. In addition, for H-SA, the band at 1108 cm^−1^ is widened. FTIR measurements for the H-APTES halloysite sample exhibit several new bands in comparison with H-NM and H-SA samples. The FTIR spectra show several bands whose intensity is mainly due to APTES: peak located at 3295 cm^–1^ due to the N–H symmetric stretching of APTES, bands at 2928 and 2885 cm^–1^ corresponded to ν_A_(C–H)_CH2_ and ν_S_(C-H)_CH3_, respectively, the band at 1483 cm^–1^ corresponded to δ(NH_2_), the siloxane stretching band, located at 1146 cm^–1^, the band lying at 1089 cm^–1^ due to the C–O symmetric stretching of APTES.

### 2.2. Adsorption Study

#### 2.2.1. Effect of Adsorbent Dose

**Figure 4:**
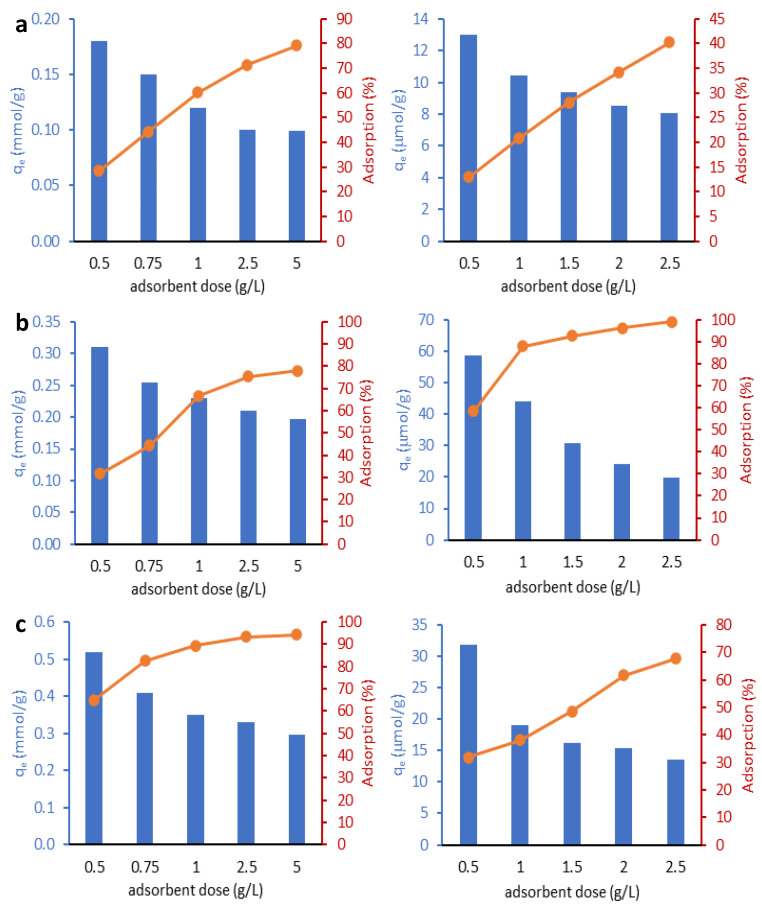
Effect of adsorbent dose on the adsorption of Cu(II) ions (left column) and RR-120 dye (right column) on the H-NM (**a**), H-SA (**b**), and H-APTES (**c**).

To determine the optimal amounts of adsorbents for adsorption tests, the effect of halloysite dosage on adsorption efficiency was investigated. For this purpose, each amount of halloysite corresponding to a dose of 0.5 to 2.5 g/L for RR-120 or 0.5 to 5.0 g/L for Cu(II) was contacted with a dye solution (50 µmol/L), and a solution containing copper(II) ions (0.787 mmol/L). Figure 4 shows the effect of varying the dose of halloysite on the adsorption efficiency of the two adsorbates from water. A similar relationship of progressive increase in adsorption with increasing dose of halloysite is observed for both adsorbates. This is due to the increase in adsorption sites available for adsorbate, which is the result of an increase in the amount of adsorbent in the solution.

When the dose of halloysites was increased from 0.5 to 5 g/L, the removal efficiency of Cu(II) ions increased from 28.6% to 79.2% for H-NM, from 65.1% to 94.3% for H-APTES and from 31.7% to 77.7% for H-SA. For the next experiments, the adsorbent dose of 2.5 g/L was used. A similar increasing trend was observed for dye adsorption. When the dose of halloysite was 2.5 g/L, the efficiency of RR120 removal was found to be 40.2% for H-NM, 67.7% for H-APTES, and reached 99% for halloysite modified with sulfuric acid. Due to the large differences in the adsorption capacity of the different halloysites towards the RR-120 dye, different doses of each adsorbent were used in further experiments—2.5 g/L for H-NM, 1.5 g/L for H-APTES and 1.0 g/L for H-SA.

#### 2.2.2. Adsorption Kinetics

**Figure 5:**
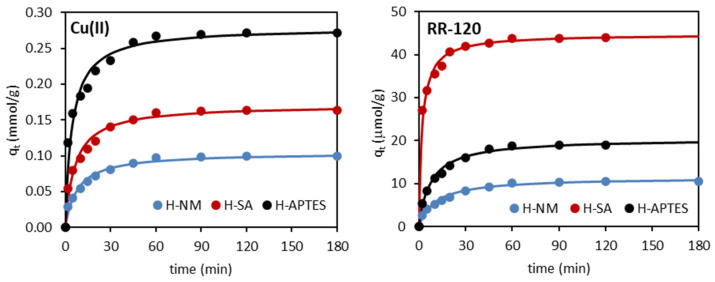
Adsorption kinetics of Cu(II) and RR-120 on the tested halloysites (line: fitting of a pseudo-second-order kinetic model).

The adsorption capacity as a function of contact time is shown in Figure 5. As can be seen, both the Cu(II) ions and the RR-120 dye were adsorbed very rapidly, with more than 80% of the initial metal load and more than 90% of the dye being adsorbed after 30 min, followed by a slow increase until equilibrium is reached after about 60 min.

The pseudo-first-order (PFO, Equation (1)) and pseudo-second-order (PSO, Equation (2)) kinetic equations were used to fit the adsorption data [21]:

(1) $$\log\left(q_{e}-q_{t}\right)=\log q_{e}-\frac{k_{1}}{2.303}t$$

(2) $$\frac{t}{q_{t}}=\frac{1}{k_{2}q_{e}^{2}}+\frac{1}{q_{e}}\;t\;$$

where *k*_1_—the pseudo-first-order adsorption rate constant (1/min); *k*_2_—the pseudo-second-order adsorption rate constant (g/mmol∙min for Cu(II) and g/µmol∙min for RR-120); and *t*—time (min).

The values of the adsorption rate constants *k*_1_ and *k*_2_, the sorption capacities *q*_e exp_ (experimental) and *q*_e cal_ (calculated), as well as the correlation coefficients *R*^2^ describing the Cu(II) and RR-120 adsorption on all three halloysites were determined by the linear regression method. The obtained values are summarized and compared in Table 5.

The choice of the equation best describing the adsorption of both adsorbates was made based on the higher values of the *R*^2^ correlation coefficients. Thus, it can be concluded that the adsorption of both copper(II) ions and RR-120 dye followed the pseudo-second-order equation (*R*^2^ ≥ 0.997). This is also confirmed by the more closely related values of the adsorption capacities experimental (*q*_e exp_) and calculated (*q*_e2 cal_) for the PSO model.

Copper(II) ions were adsorbed best on H-APTES and weakest on unmodified halloysite (H-NM < H-SA < H-APTES). The values of the adsorption rate constants *k*_2_ increased in the order: H-APTES < H-SA < H-NM.

Adsorption of RR-120 dye was most efficient on halloysite H-SA (*q*_e_ = 44.84 μmol/g), less efficient on halloysite H-APTES (*q*_e_ = 19.05 μmol/g), and least efficient on halloysite H-NM (*q*_e_ = 11.42 μmol/g). The adsorption of the dye on halloysites was fastest on halloysite H-SA (*k*_2_ = 0.0104 g/(μmol∙min)), slower on H-NM (*k*_2_ = 0.0083 g/(μmol∙min)) and slowest on H-APTES (*k*_2_ = 0.0064 g/(μmol∙min)), so that, in contrast to Cu(II) ions, it correlated with the mesopore volume of each adsorbent. The specific adsorption takes place mainly in the micropores and partly in the mesopores. Thus, in general, the large volume of the micropores of an adsorbent is reflected in its large adsorption capacity. Mesopores also participate in adsorption but their primary function is transport—they are the “road/highway” by which the adsorbate is transported to the micropores. A well-developed mesoporous structure usually translates into a better kinetic capacity of the adsorbent (higher rate of adsorbate uptake in solution). The observed correlation of the adsorption rate of the dye with the mesopore volume of individual halloysites suggests that its adsorption depends more on the porous structure of the adsorbent than on the functionalization of its surface. In the case of Cu(II) ions, no correlation between adsorption and the porous structure of the adsorbents was observed, suggesting a completely different mechanism of adsorption of metal ions compared to dye molecules.

#### 2.2.3. Adsorption Isotherms

**Figure 6:**
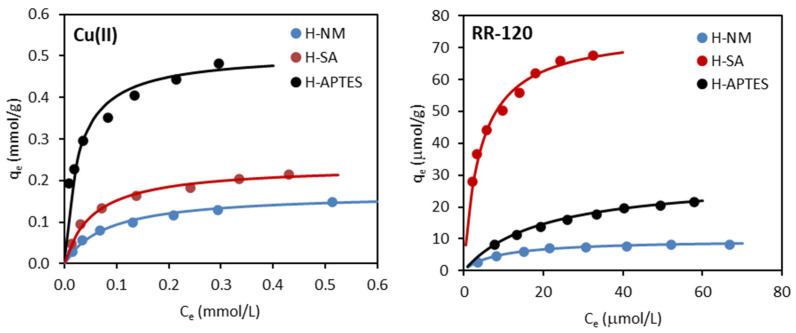
Adsorption isotherms of Cu(II) ions and RR-120 dye on the halloysite samples (line: fitting of Langmuir isotherm).

The adsorption isotherms of the copper(II) ions and the RR-120 dye on H-NM, H-SA, and H-APTES from aqueous solutions are shown in Figure 6. Three isotherm models, namely the Freundlich, Langmuir, and Temkin, were used in this study to describe the adsorption data [22]. 

The Freundlich model assumes multilayer adsorption on heterogeneous and non-uniform surfaces and is given by Equation (3).

(3) $$q_{e}=K_{F}C_{e}^{1/n}$$

where *K*_F_ ((mmol/g)(L/mmol)^1/n^ for Cu(II) and (µmol/g)(L/µmol)^1/n^ for RR-120) and *n* are the Freundlich constants.

The Langmuir model is based on the assumption of monolayer adsorption on homogeneous surfaces and is expressed by Equation (4)

(4) $$q_{e}=\frac{q_{m}K_{L}C_{e}}{1+K_{L}C_{e}}$$

where *q*_m_ is the maximum adsorption capacity (mmol/g form Cu(II) and µmol/g for RR-120) and *K*_L_ is the Langmuir constant (L/mmol or L/µmol for Cu(II) or RR-120, respectively).

The Temkin isotherm, which assumes that the free energy of adsorption is a function of the adsorbent surface coverage and also takes into account the effect of intermediate adsorbate–adsorbate interactions on the adsorption process, can be expressed by the following equation:

(5) $$q_{e}=\frac{RT}{b_{T}}ln(A_{T}C_{e})$$

where *b*_T_ is the Temkin isotherm constant related to the heat of adsorption (J/mol); *A*_T_ is the equilibrium binding constant corresponding to the binding energy (L/g); *R* is the gas constant (8.314 J/mol·K); and *T* (K) is the absolute temperature.

The calculated parameters of the Freundlich, Langmuir, and Temkin isotherms describing the adsorption of Cu(II) ions and the dye RR-120 on halloysites, along with the correlation coefficients *R*^2^, are summarized in Table 6.

The data presented in Table 6 indicate that all three models describe the adsorption of copper(II) ions and the dye Reactive Red 120 from aqueous solutions on halloysites quite well. Comparing the correlation coefficient *R*^2^, it can be seen that the Langmuir model best describes the adsorption of both adsorbates on halloysites, suggesting monolayer adsorption of both adsorbates on homogeneous halloysite surfaces. Comparing the calculated *q*_m_ and *K*_F_ parameters, it can be observed that both modifications of halloysite (with sulfuric acid and (3-aminopropyl)triethoxysilane) significantly improved their adsorption capacities. However, the effect of modification on adsorption was different for metal ions and dye.

The *q*_m_ values calculated from the Langmuir model for Cu(II) ions were 0.169 mmol/g for H-NM, 0.236 mmol/g for H-SA, and 0.507 mmol/g for APTES-modified halloysite, respectively. The adsorption capacity of H-APTES was ~3.0 times higher than that of H-NM and ~2.1 times higher than that of H-SA, although this adsorbent had the lowest BET-specific surface area (Table 2). The adsorption capacity of sulfuric acid-activated halloysite increased ~1.4 times compared to unmodified halloysite although its BET surface area increased significantly from 53 to 129 m^2^/g. These results indicate that the NH_2_ functionalization, rather than the specific surface area, was responsible for the enhanced adsorption of copper ions on the halloysite. These observations are generally in agreement with results reported by other authors who studied the adsorption of copper ions on various materials functionalized with amine groups. The presence of surface –NH_2_ groups improved Cu(II) adsorption on, inter alia, modified MCM-41 [23], APTES-modified zeolite [24], amino-functionalized mesoporous silica [25], or magnetic nanoparticles [26].

The mechanism for the adsorption of Cu(II) ions on amino-functionalized materials includes surface complexation, electrostatic attraction, and ion exchange of amino hydrolysis by H-bonding between amino and hydroxyl groups, as proposed by Li et al. [26]. In solution, at the natural pH (~5–6), –NH_2_ groups on the surface can undergo protonating and deprotonating reactions (Equation (6)).

−NH_2_ + H^+^ ↔ –NH_3_^+^ (6)

At the same time, complexes of Cu^2+^(CuOH^+^) with the amino groups on the adsorbent surface are formed via coordination interactions (Equations (7) and (8)).

−NH_2_ + Cu^2+^ ↔ –NH_2_Cu^2+^ (7)

−NH_2_ + Cu^2+^ + H_2_O ↔ –NH_2_Cu(OH)^+^ + H^+^ (8)

Furthermore, an ion exchange between the amino groups and the hydroxyl groups from the solution takes place via hydrogen bonds (Equation (9)) and so formed −NH_2_OH^−^ groups can react with Cu^2+^(CuOH^+^) via electrostatic attraction and/or surface complexation according to Equation (10).

−NH_2_ + H_2_O ↔ –NH_2_OH^−^ + H^+^ (9)

−NH_2_OH^−^ + Cu^2+^(CuOH^+^) ↔ −NH_2_OH^−^····Cu^2+^(−NH_2_OH^−^····CuOH^+^) (10)

As one might guess, the adsorption of copper(II) ions on APTES-modified halloysite probably follows the same mechanism.

To fully assess the suitability of the materials used for the removal of copper ions, it is necessary to compare them with other adsorbents. Such a comparison is shown in Table 7, where the adsorption capacities of various other mineral adsorbents (raw and modified) are listed. For comparison, the *q*_m_ values obtained from the Langmuir model were used. These values were converted to the same units (mg/g) where necessary. The adsorption capacities of other non-mineral adsorbents can be found in the review paper [27]. The data in Table 7 show that the adsorption capacities of the halloysites used in this work are very good.

The RR-120 dye was also better adsorbed on modified halloysites than on H-NM, although in contrast to Cu(II), its adsorption was most effective on sulfuric acid-modified halloysites. This suggests that, unlike copper ions, the adsorption of RR-120 dye is determined by the textural properties (specific surface area) of the adsorbent rather than by chemical modification of its surface. This is supported by the results presented in Section 3.1. The FTIR spectra (Figure 3 and Table 4) for H-SA are almost identical to those for H-NM, indicating that the modification of halloysite with sulphuric acid has not changed the chemical character of its surface. The TG results (Table 3) also confirm this. The only observable change is a significant increase in the porosity of H-SA, including an increase in its specific surface area. The enhanced adsorption of RR-120 on H-SA is, therefore, the result of its higher BET surface area and, therefore, more active sites to which the dye molecules can attach.

The adsorption capacity determined from the Langmuir model was 9.64 μmol/g for the original halloysite, 29.33 μmol/g for H-APTES, and 75.76 μmol/g for H-SA. Thus, the activation of halloysite with sulfuric acid was the most favorable, significantly increasing its specific surface area from 53 m^2^/g (H-NM) to 129 m^2^/g (H-SA), as shown in Table 2. The increase in adsorption capacity of H-SA is, therefore, most likely due to its increased BET surface area and thus more active sites/centers available for the adsorbate molecules. Modification of halloysite with (3-aminopropyl)triethoxysilane also significantly increased the adsorption capacity of the resulting material compared to the original mineral. The adsorption capacity of H-APTES increased even though its specific surface area decreased as a result of the modification (from 53 m^2^/g for the original halloysite to 34 m^2^/g), indicating a different mechanism of dye adsorption on this material compared to H-SA.

The removal of dyes from aqueous solutions using various adsorbents is a very complex and demanding process. Specific adsorption mechanisms include van der Waals interactions, ion exchange, electrostatic interactions, surface complexation, hydrophobic and π-π interactions, or hydrogen bond formation [39]. In the case of H-SA, the improvement in its adsorption capacity is accompanied by an increase in its BET surface area. This suggests that the adsorption of RR-120 on its surface is mainly due to pore filling and hydrophobic, π-π, and hydrogen bonding interactions. The enhanced adsorption properties of halloysite H-APTES are mainly due to the presence of basic groups (–NH_2_) derived from (3-aminopropyl)triethoxysilane on its surface. The surface amine groups are critical for anionic binding (in this case the anionic dye RR-120) and H-bond formation. Adsorption studies were carried out in solutions of the dye at a natural pH (~6.1). The mechanism of adsorption of the anionic dye in an acidic environment (pH 6.1) is related to the protonation of the amine groups of the adsorbent, as shown by the following reaction:

Halloysite–NH_2_ + H^+^ ↔ Halloysite–NH_3_^+^ (11)

At the same time, the dye molecule dissociates:

RR-120–SO_3_Na → RR-120–SO_3_^−^ + Na^+^ (12)

As a result of the electrostatic interaction between the adsorbent and the adsorbate molecule, adsorption takes place according to the equation:

Halloysite–NH_3_^+^ + RR-120–SO_3_^−^ ↔ Halloysite–NH_3_^+^–SO_3_^−^–RR-120 (13)

Table 8 compares the adsorption capacity of the halloysites with other raw and modified minerals reported in the literature. The adsorption capacities of the halloysites described in this paper are satisfactory. A comparison with other non-mineral adsorbents can be found in a recently published review paper [39].

#### 2.2.4. Effect of Solution pH

The physicochemical properties of the solution, and in particular its pH, play an important role in the adsorption process as they affect the degree of ionization of the adsorbate (metal speciation) and also the charge that can appear on the surface of the adsorbent. An important parameter for determining the surface charge on the adsorbent is the pH point of zero charge (pH_pzc_). Thus, in a solution with a pH < pH_pzc_, a positive charge accumulates on the surface, whereas in a solution with a pH > pH_pzc_, the surface of the adsorbent is negatively charged. The pH_pzc_ values for all three halloysite samples were determined using the pH drift method as described elsewhere [49] and were found to be 6.5 for H-NM, 6.9 for H-APTES, and 4.3 for H-SA.

The effect of the initial pH on the adsorption capacity of halloysites for Cu(II) was studied by changing the pH of the solution from 2 to 6, while for the RR-120 dye for solutions with pH in the range of 2–10 (pH of the solutions was adjusted with 0.01 mol/L NaOH and/or 0.01 mol/L HCl). Such a limited range of measurement was applied to copper ions since Cu^2+^ precipitation occurs at a pH higher than 6.0 [26,34,50].

**Figure 7:**
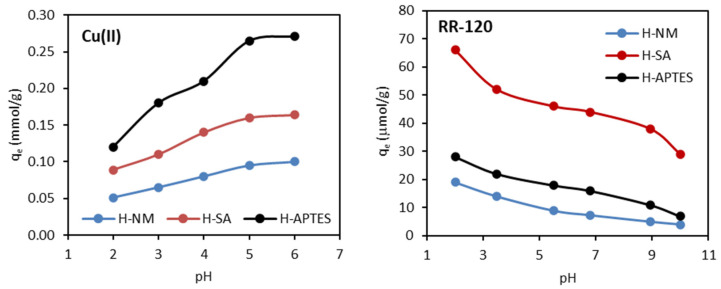
Effect of initial pH on the adsorption of Cu(II) ions and RR-120 dye on halloysite samples.

The adsorption capacity of Cu(II) and RR-120 dye on halloysite samples at different pH is shown in Figure 7. 

It can be seen that the adsorption of Cu(II) is strongly pH-dependent and increases with an increase in pH. As the pH increased from 2.0 to 6.0, the adsorption capacity of Cu(II) on H-NM, H-SA, and H-APTES increased from 0.051 to 0.101 mmol/g, from 0.089 to 0.164 mmol/g and from 0.120 to 0.271 mmol/g, respectively. The adsorption capacity was very low in a strongly acidic environment and increased with increasing pH values until it reached optimal efficiency in the pH range of 5.0–6.0. The relatively low adsorption in acidic media is probably due to the competition of positively charged H^+^ ions with Cu^2+^ ions for the same adsorption sites and functional groups present on the adsorbent surface [50]. For example, in the case of H-APTES, hydrogen ions present in solution in large quantities competed with Cu(II) cations for ion exchange with NH_4_^+^ ions derived from APTES. As reported by Zhang et al. [51], H^+^ ions may also inhibit the dissociation of protons from other surface functional groups (e.g., carboxyl and hydroxyl groups) necessary for ion exchange with Cu(II) cations. The observed successive increase in adsorption efficiency with increasing pH can, therefore, be explained by a decrease in competition between H^+^ and Cu^2+^ ions for the same surface groups. Furthermore, with increasing pH, the number of negatively charged functional groups on the adsorbent surface also increases, which, as suggested by Rahman and Islam [50], increases the adsorption of positively charged cupric ions. As suggested by other authors [26,50], copper ions in solution at pH 6.0 can exist in three species (Cu^2+^, Cu(OH)^+,^ and Cu(OH)_2_), which can be adsorbed by ion exchange mechanism or by hydrogen bonding. A similar trend in the adsorption of copper(II) ions—an increase in adsorption efficiency with increasing pH—was reported by other authors, among others, on the maple wood sawdust [50], amino-functionalized magnetic nanoparticles [26], maghnite [34], poly(acrylic acid/chestnut shell pigment) hydrogel [51], amine-functionalized MCM-41 [23] or natural zeolite modified with APTES [24].

The effect of pH on the adsorption of RR-120 dye from aqueous solutions was studied in the pH range of 2 to 10 as is shown in Figure 7. It can be seen that the adsorption of RR-120 dye on halloysites is closely related to the initial pH of the solution. The adsorption capacity of RR-120 dye decreases as the pH of the solution increases from 2 to 10. For all three adsorbents, the maximum adsorption was found at pH 2, when 43, 56, and 68% of the initial amount of dye was adsorbed on H-NM, H-APTES, and H-SA, respectively. The RR-120 is an anionic dye and contains six sulfonate groups in its molecule, the pKa of which is 2.1 [52]. This means that it exists in the anionic form in practically the entire pH range over which the experiments were carried out (2–10), due to the transformation of the –SO_3_H group into a negative –SO_3_^–^ group in an aqueous medium. The pH effect on RR-120 adsorption can be explained by the pH_pzc_ concept. The H-NM, H-APTES, and H-SA had different values of pH_pzc_ as 6.5, 6.9, and 4.3, respectively. Consequently, the halloysites obtain a positively charged surface at the solution pH below pH_pzc_, which favors the adsorption of an anionic dye such as RR-120 due to electrostatic attraction. The decrease in adsorption at high solution pH (pH > pH_pzc_) results from a stronger electrostatic repulsion between the negatively charged surface of the halloysite and the anionic dye molecule. Similar results showing a decrease in adsorption with increasing pH were reported by other researchers [20,52,53,54,55,56,57,58,59].

## 3. Materials and Methods

### 3.1. Reagents and Materials

The Reactive Red 120 azo dye (RR, C_44_H_24_Cl_2_N_14_Na_6_O_20_S_6_, CAS number: 61951-82-4) was purchased from Boruta-Zachem SA (Bydgoszcz, Poland), the (3-aminopropyl)triethoxysilane (APTES) was obtained from Sigma-Aldrich (St. Louis, MO, USA) while cuprizone (bis-(cyclohexanone) oxaldehydrozone)–synthetic chelating compound used for the determination of copper(II) ions was purchased from Acros Organics (Geel, Belgium). Other high-purity reagents including copper(II) sulfate pentahydrate (CuSO_4_·5H_2_O) were received from Chempur (Piekary Śląskie, Poland). The halloysite samples were received from the “Dunino” mine (Intermark Company, Legnica, Poland). The morphology and structure of this raw mineral were described elsewhere [60,61].

### 3.2. Preparation and Characterization of Adsorbents

First, the native halloysite (H-NM) was mixed with ethanol in a weight ratio of 1:3, then it was subjected to ultrasound (35 kHz) for 3 h in an ultrasound bath IS-7S (Intersonic S.C., Olsztyn, Poland), and at the same time, it was stirred with a mechanical stirrer. After this time, APTES was added to the mixture in an amount corresponding to 15% of the mass of halloysite and the process was continued for another 3 h under the same conditions as before. After the modification, the solvent was evaporated and the obtained product (H-APTES) was ground to a powder. Drying took place at 90 °C.

The native halloysite was also treated with sulfuric acid (98%) in proportion 92:8. The first stage involved the preliminary treatment of native halloysite fraction with ultrasounds of frequency 30 kHz for 2–4 h in an ultrasounds bath. The obtained product was separated from the unreacted reagent to obtain the final modified halloysite (H-SA) and then dried at 90 °C.

For both modified samples (H-APTES and H-SA) and also for native halloysite (H-NM) the Brunauer–Emmett–Teller (BET) specific surface areas as well as micro- and mesopore volumes were calculated based on measured low-temperature nitrogen adsorption isotherms using a TriStar II 3020 V1.03 (Micromeritics Company, Norcross, GA, USA).

Determination of their surface chemical compositions was carried out using JSM-6490LV (JEOL Company, Peabody, MA, USA) scanning electron microscope coupled with an energy dispersive X-ray spectrometer (EDS).

Thermogravimetric analysis was performed using a thermobalance type TGA Q50 (Thermo Scientific, Waltham, MA, USA). The measurement was carried out at the heating rate of 10 °C/min in the temperature regime from 25 up to 800 °C in an inert gas (nitrogen) atmosphere.

For halloysite samples, the surface chemistry was additionally characterized by recording FTIR spectra using a Nicolet iS10 FTIR spectrometer (Thermo Scientific, Waltham, MA, USA).

### 3.3. Bath Adsorption Experiments

Adsorption studies of Cu(II) ions and Reactive Red 120 dye on all three halloysites were performed in Erlenmeyer flasks containing adsorbate solutions of appropriate concentrations and corresponding adsorbent weights. The flasks were shaken at a constant speed of 100 rpm at room temperature. All experiments were repeated twice and the average of the two measurements was used for further calculations. Adsorption kinetics and equilibrium adsorption (adsorption isotherms) were studied as well as the effects of adsorbent dose and solution pH.

The experimental procedures varied due to the individual characteristics of the metal ions and the dye. For example, for the adsorption of copper(II) ions, the solution volume was always 20 mL, and the initial concentrations ranged from 0.157 to 1.259 mmol/L (10 to 80 mg/L). Kinetic studies and experiments on the effect of adsorbent dose and solution pH were performed for Cu(II) solutions with initial concentrations of 0.787 mmol/L (50 mg/L). For the RR-120 dye, solution volumes were 10 mL and initial concentrations ranged from 10 to 100 µmol/L. The effect of adsorbent dosage, solution pH, and contact time on adsorption was performed for an initial dye concentration of 50 µmol/L.

The natural (original) pH of the adsorbate solutions (~5.7 for Cu(II) and ~6.1 for RR-120) was chosen for the adsorption studies.

The concentrations of both adsorbates in the solutions after adsorption were measured using a UV–Vis spectrophotometer (Varian Carry 3E series, Palo Alto, CA, USA). Copper ions were determined spectrophotometrically in the form of complexes with cuprizone, which forms a blue chelate with copper in a slightly alkaline environment. The Cu(II) ion concentration was measured at a wavelength of 600 nm corresponding to the absorption maximum of the complex formed. The calibration curve for the determination of copper(II) ions was linear (R^2^ = 0.999) over the tested concentration range of 0.0016 to 0.0472 mmol/L (0.1–3.0 mg/L) and was described by the equation y = 15.563x + 0.0089. The concentration of RR-120 dye in the solutions was determined spectrophotometrically by measuring the absorbance at 512 nm. The resulting calibration curve (y = 0.0331x + 0.011) was linear over the entire range studied, from 1 to 100 µmol/L (R^2^ = 0.999).

The adsorption efficiency (%), as well as the amount of adsorbate adsorbed at equilibrium (*q*_e_) and at time t (*q*_t_), were calculated by the following equations

(14) $$Adsorption\left(\%\right)=\frac{\left(C_{0}-C_{e}\right)}{C_{0}}\times 100$$

(15) $$q_{t}=\frac{(C_{0}-C_{t})V}{m}$$

(16) $$q_{e}=\frac{(C_{0}-C_{e})V}{m}$$

where *V* is the volume of the solution (L), *m* is the mass of the halloysite sample (g), while *C*_0_, *C*_t,_ and *C*_e_ are the initial adsorbate concentration, concentration after time *t,* and concentration at equilibrium, respectively. The concentrations of Cu(II) ions are expressed in mmol/L, while the concentrations of the RR-120 dye are expressed in µmol/L.

## 4. Conclusions

In this paper, the adsorption of copper(II) ions and Reactive Red 120 azo dye (RR-120) as models of water contaminants on unmodified (H-NM) and modified halloysites was studied. Both (3-aminopropyl)triethoxysilane (H-APTES) and sulfuric acid (H-SA) were used to modify the halloysite. Adsorption kinetics, adsorption isotherms as well as the effects of adsorbent dosage and solution pH were investigated. The results demonstrated that the adsorption of both the adsorbates increased with the increase in halloysite dosage. Adsorption was strongly pH-dependent and increased (Cu(II)) or decreased (RR-120) with an increase in solution pH. The adsorption kinetics followed the pseudo-second-order model. Adsorption isotherms were well described by the Langmuir model. A maximum capacity of Cu(II) adsorbed on natural halloysite as well as on H-SA and H-APTES at equilibrium was 0.169, 0.236, and 0.507 mmol/g, respectively. The maximum adsorption capacities for the RR-120 dye were 9.64, 75.76, and 29.33 μmol/g for H-NM, H-SA, and H-APTES, respectively. Both modification methods increased the adsorption of the copper(II) ions as well as the RR-120 dye. The results suggest that Cu(II) adsorption is rather affected by the surface chemistry of the halloysite, while the porous structure (specific surface area) of the adsorbent plays a major role in the adsorption of the dye. The results showed that APTES-functionalization and activation by sulfuric acid are promising modifications, and both modified halloysites have good application potential for heavy metals as well as azo dye removal.

## Figures and Tables

**Table 1:** The EDS analysis results of the halloysite samples.

Adsorbent	C	O	Al	Si	Fe	Ti	N
	at.%						
H-NM	-	63.5	16.0	18.4	1.8	0.3	-
H-SA	-	63.2	15.7	19.2	1.7	0.2	-
H-APTES	28.9	36.9	12.9	16.7	0.9	0.4	3.3

**Table 2:** Specific surface area and pore volumes of the halloysite samples.

Adsorbent	*S*_BET_ (m^2^/g)	*V*_t_ (cm^3^/g)	*V*_mi_ (cm^3^/g)	*V*_me_ (cm^3^/g)
H-NM	53	0.217	0.019	0.198
H-SA	129	0.399	0.057	0.342
H-APTES	34	0.108	0.0147	0.093

**Table 3:** Thermal properties of the row and modified halloysite samples.

	H-NM		H-SA		H-APTES	
	Temp. (°C)	Δm (%)	Temp. (°C)	Δm (%)	Temp. (°C)	Δm (%)
End of drying	180	2.1	200	2.6	185	9.5
Temp. of max of DTG	448	7.2	459	9.5	472	19.5
End of weight loss	680	9.4	700	11.6	715	25.8

**Table 4:** FTIR analysis of raw and modified halloysite samples.

H-NM	H-SA	H-APTES
Wavenumber (cm^−1^)		
3697 3622 1634 1108 913 791 753	3696 3621 1634 1107 911 791 752	3697 3622 3295 2930 2886 1631 1484 1140 1108 1089 913 791 753

**Table 5:** Kinetic modeling data for the adsorption of Cu(II) ions and RR-120 dye on halloysite samples.

Kinetic Model	Adsorbent/Adsorbate		
	H-NM	H-SA	H-APTES
		**Cu(II)**	
*q*_e exp_ (mmol/g)	0.101	0.164	0.271
Pseudo-first-order			
*k*_1_ (1/min)	0.0420	0.0428	0.0490
*q*_e1 cal_ (mmol/g)	0.051	0.104	0.151
*R* ^2^	0.948	0.966	0.986
Pseudo-second-order			
*k*_2_ (g/mmol∙min)	1.211	0.913	0.790
*q*_e2 cal_ (mmol/g)	0.105	0.171	0.279
*R* ^2^	0.999	0.999	0.998
		**RR-120**	
*q*_e exp_ (µmol/g)	10.42	43.96	19.03
Pseudo-first-order			
*k*_1_ (1/min)	0.0843	0.0615	0.0608
*q*_e1 cal_ (µmol/g)	15.73	25.01	17.87
*R* ^2^	0.905	0.911	0.921
Pseudo-second-order			
*k*_2_ (g/µmol∙min)	0.0083	0.0104	0.0064
*q*_e2 cal_ (µmol/g)	11.42	44.84	19.05
*R* ^2^	0.997	0.999	0.999

**Table 6:** The Freundlich, Langmuir, and Temkin isotherm constants for the adsorption of Cu(II) ions and RR-120 dye on halloysite samples.

Isotherm Model	Adsorbent/Adsorbate		
	H-NM	H-SA	H-APTES
		**Cu(II)**	
Freundlich			
*K*_F_ ((mmol/g)(L/mmol)^1/n^)	0.230	0.329	0.675
1/*n*	0.449	0.397	0.264
*R* ^2^	0.943	0.944	0.990
Langmuir			
*q*_m_ (mmol/g)	0.169	0.236	0.507
*K*_L_ (L/mmol)	12.50	18.16	39.01
*R* ^2^	0.995	0.996	0.995
Temkin			
*b*_T_ (kJ/mol)	72.87	53.39	29.78
*A*_T_ (L/g)	151.5	233.9	966.6
*R* ^2^	0.961	0.965	0.921
		**RR-120**	
Freundlich			
*K*_F_ ((µmol/g)(L/µmol)^1/n^)	1.750	24.04	3.140
1/*n*	0.406	0.316	0.487
*R* ^2^	0.941	0.973	0.987
Langmuir			
*q*_m_ (µmol/g)	9.640	75.76	29.33
*K*_L_ (L/µmol)	0.111	0.241	0.051
*R* ^2^	0.999	0.997	0.997
Temkin			
*b*_T_ (kJ/mol)	1.198	0.169	0.361
*A*_T_ (L/g)	1.047	3.406	0.400
*R* ^2^	0.989	0.992	0.994

**Table 7:** Comparison of the adsorption capacities of halloysites with some other mineral adsorbents for Cu(II) adsorption.

Adsorbent	*q*_m_ (mg/g)	Ref.
H-NM	10.73 *	This paper
H-SA	14.98 *	This paper
H-APTES	32.19 *	This paper
alginate/kaolin	0.339	[28]
bentonite clay	0.406	[29]
alginate/montmorillonite	0.680	[28]
clinoptilolite	5.269	[30]
zeolite modified with APTES	6.176	[24]
natural montmorillonite	12.22	[31]
palygorskite	12.53	[32]
urea modified halloysite (H-Mo)	13.87	[17]
melamine modified halloysite (H-Me)	15.24	[17]
H_2_SO_4_-activated montmorillonite	15.40	[31]
kaolinite	16.79	[33]
maghnite	21.78	[34]
natural smectite	28.82	[35]
natural Ca-bentonite	32.26	[36]
vermiculite	32.68	[32]
CH_3_COONa-intercalated halloysite	52.30	[16]
CaCl_2_ pretreated Algerian bentonite	55.47	[37]
acid-activated palygorskite	93.02	[38]

* converted from mmol/g.

**Table 8:** Comparison of the adsorption capacities of halloysites with some other mineral materials for the adsorption of Reactive Red 120.

Adsorbent	*q*_m_ (mg/g)	Ref.
H-NM	12.90 *	This paper
H-SA	101.4 *	This paper
H-APTES	39.24 *	This paper
pumice	0.320	[40]
kaolin	1.090	[41]
chitosan-montmorillonite composite	5.608	[42]
natural untreated clay (Tunisia)	29.94	[43]
CTAB-modified bentonite	51.28	[44]
raw Fouchana clay	54.64	[45]
CTAB-modified bentonite	81.97	[46]
zeolite	145.9	[47]
Fe_3_O_4_@zeolite composite	154.3	[47]
HDTMA-bromide modified clay	163.9	[45]
functionalized magnesium phyllosilicate	229.9	[48]

* converted from μmol/g.

## Data Availability

The data presented in this study are available on request from the corresponding author.
